# Porcine Fetal Hypothyroidism Induces Temporal and Tissue‐Specific Alterations in the Insulin‐Like Growth Factor System

**DOI:** 10.1002/cph4.70028

**Published:** 2025-07-22

**Authors:** Alyssa A. Smith, J. Alex Pasternak

**Affiliations:** ^1^ Department of Animal Sciences Purdue University West Lafayette Indiana USA

**Keywords:** fetal, hypothyroidism, IGFBP, insulin‐like growth factor, methimazole, thyroid hormone

## Abstract

To characterize the interaction between the fetal thyroid and IGF system, we utilized a temporal model of porcine fetal hypothyroidism. *N* = 24 pregnant gilts were split into two treatment groups, with 12 gilts receiving a daily oral dose of 5 mg/kg methimazole (MMI) to induce fetal hypothyroidism, and 12 gilts serving as a control. Within each group, three gilts began treatment at gestational Days 34, 45, 55, or 65 of gestation, with gilts euthanized 21 days later to allow for fetal sample collection. Fetuses from the MMI groups were confirmed hypothyroid by the presence of goiter, as well as reduced circulating thyroxine levels at all four timepoints, and reduced circulating triiodothyronine levels at the two later timepoints. Samples of fetal liver and kidney were taken from four fetuses per litter for assessment of gene expression, and IGFBP levels were also assessed in serum. Among control fetuses, many of the IGFBPs were temporally regulated throughout gestation in the liver, kidney, and serum, with the expression of *IGF1* also temporally regulated. Within each timepoint, temporal downregulations in the expression of hepatic *IGFBP1*, ‐*2*, ‐*3*, ‐*4*, ‐*6*, and ‐*7*, as well as renal *IGFBP3* and ‐*7* were observed in MMI fetuses, with no changes in the expression of either IGF ligand. In contrast, MMI treatment resulted in minimal changes in sera IGFBP levels. These results suggest a temporal and tissue‐specific relationship between thyroid hormones and the IGF system in the fetal pig, but provide little evidence of systemic disruptions in the IGF system in response to fetal hypothyroidism.

## Graphical abstract

Fetal hypothyroidism has detrimental impacts on growth and development. Here we demonstrate that hypothyroidism causes temporal dysregulations in insulin‐like growth factor binding protein expression in the fetal liver and kidney, although these effects are not reflected systemically, suggesting a tissue‐specific response that may mediate localized adverse effects of hypothyroidism.

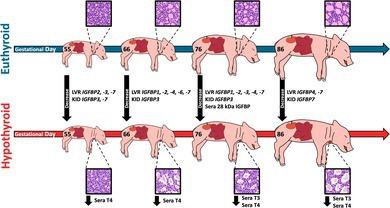

## 1 Introduction

Throughout gestation, the fetus undergoes a wide array of ontogenic changes in critical endocrine systems needed for proper in utero growth and development. Among these critical endocrine systems is the thyroid system, with key hormones including thyroxine (T4) and its more bioactive derivative 3,5,3′‐triiodothyronine (T3) not only substantially upregulated in gestating females (Brent 1999), but also increasing in concentration in the fetus throughout gestation (Allen et al. 1998; Brzezińska‐Slebodzińska and Slebodziński 2004; Devaskar et al. 1986). This increase in activity within the maternal and fetal hypothalamic–pituitary–thyroid (HPT) axis is susceptible to various perturbations, the most common of which is a deficit in thyroid hormone production (hypothyroidism). In humans, congenital hypothyroidism is the most common neonatal endocrine disorder (Ghirri et al. 2016), affecting between 1:2000 and 1:4000 infants and resulting in postnatal consequences including non‐allometric growth, delayed bone development (Rastogi and LaFranchi 2010), and mental retardation (Oerbeck et al. 2003). Swine are also susceptible to fetal hypothyroidism in response to in utero viral infection (Pasternak et al. 2020a), which is of further importance to the agricultural industry. In many species, the conceptus becomes increasingly thyroid‐independent starting early in development, following the establishment of a placental‐enzymatic barrier that prevents transmission of bioactive maternal thyroid hormones (Krysin et al. 1997; Roti et al. 1981). As a result, dysfunction of the fetal thyroid gland at any gestational timepoint poses a significant threat to normal fetal physiology.

Prior research has suggested an interaction between the HPT axis and other endocrine systems critical for fetal development. This includes an interaction with the insulin‐like growth factor (IGF) system, creating the potential for secondary endocrine disruption in response to hypothyroidism. The IGF system is a vital regulator of both fetal and postnatal growth and is canonically recognized to mediate the effects of growth hormone. The two key IGF ligands include insulin like growth factor 1 (IGF1) and insulin like growth factor 2 (IGF2), the latter of which may be secreted independently of growth hormone (Mesiano et al. 1989) and is substantially upregulated during fetal life (Gluckman and Butler 1983; Näntö‐Salonen et al. 1991; Pan et al. 2012). The bioactivity of the IGFs is achieved by binding to the insulin like growth factor 1 receptor (IGF1R), which is ubiquitously expressed in almost all tissues and has a higher affinity for IGF1 than IGF2 (Hakuno and Takahashi 2018). Ligand bioavailability is also regulated by a second receptor (IGF2R), which internalizes and degrades IGF2, effectively preventing it from acting on the IGF1R. Further, over 90% of circulating IGFs exist bound to a family of insulin like growth factor binding proteins (IGFBPs), which serve many functions including inhibiting IGFs from interacting with their receptors and also greatly prolonging the half‐lives of circulating IGFs (Guler et al. 1989; LeRoith et al. 2021). While the number of IGFBP genes and transcripts varies across species, both pigs and humans possess six high‐affinity IGFBPs (IGFBP1‐6) (Allard and Duan 2018), with a number of low‐affinity binding proteins, including IGFBP7, also contributing to IGF binding (Hwa et al. 1999).

The effects of hypothyroidism on the fetal and neonatal IGF system have been well documented in thyroidectomized fetal sheep (Camm et al. 2021; Forhead et al. 1998; Forhead et al. 2002) and antithyroid drug‐treated rodents (Näntö‐Salonen et al. 1991; Elder et al. 2000; Näntö‐Salonen and Rosenfeld 1992). While the exact mechanism by which these systems interact is unclear, some effects appear directly mediated by thyroid hormones, while others appear reliant on indirect mechanisms (Näntö‐Salonen et al. 1993). Only a handful of prior studies have examined the relationship between thyroid hormones and the IGF system in fetal swine under abnormal endocrine conditions (Hausman et al. 2000; Latimer et al. 1993; Spencer et al. 1989), collectively suggesting a complex relationship that is dependent on tissue, gestational age, and target. However, a more comprehensive understanding of this interaction across a wider range of targets and gestational timepoints is warranted to further elucidate the impact of hypothyroidism on the IGF system in the fetal pig. Of dual significance, this analysis would not only hold value for providing insights into the consequences of thyroid hormone disruption in virally infected swine fetuses, but also help to inform the utility of the pig as a biomedical model for studying the impacts of fetal hypothyroidism.

To this end, the objective of the current study was to characterize the temporal and tissue‐specific relationship between thyroid hormones and the IGF system in the fetal pig, with a focus on the fetal liver (LVR) and kidney (KID). The LVR was selected as it is the primary source of both IGFs and IGFBPs, while the expression of IGFs in the porcine fetal KID under abnormal endocrine conditions has previously been shown to be temporally dependent in late gestation (Latimer et al. 1993). As our prior work in the late gestation fetal pig reported a minimal impact of hypothyroidism on relative organ growth, skeletal development, and tissue protein accretion (Ison et al. 2023; Smith et al. 2024), we hypothesized that the detrimental impact of hypothyroidism on the IGF system would originate earlier in gestation, with this system exhibiting temporal and tissue‐specific responses to fetal hypothyroidism. To assess this, we utilized a gestational‐age dependent model of porcine fetal hypothyroidism applied at 10‐day intervals throughout gestation, with the earliest treatment period beginning prior to the onset of fetal thyroid function and the latest timepoint terminating at the start of late gestation. Fetal hypothyroidism was confirmed via a combination of thyroid histology and measurement of circulating T3 and T4, and the temporal response of the fetal IGF system to hypothyroidism was assessed via analysis of changes in relative hepatic and renal gene expression of the major IGF ligands and binding proteins. Subsequently, the impact of tissue dysregulations on the systemic IGF system was assessed via measurement of IGFBP levels in serum.

## 2 Materials and Methods

### 2.1 Animal Model

Twenty‐four commercial crossbred gilts (Yorkshire × Landrace) were selected from and housed individually at the Purdue University Animal Sciences Research and Education Center. A daily oral dose of 17.6 mg Altrenogest (Merck Animal Health, Kenilworth, NJ, USA) was administered in feed for 14 days to synchronize estrous, after which the gilts were bred via artificial insemination with pooled Duroc semen in the first standing estrus following withdrawal. Gilts were confirmed pregnant via transcutaneous ultrasound around gestational day (GD) 30, before evenly dividing the maternal population over four gestational timepoints, with *n* = 6 gilts assigned to receive a 21‐day course of treatment beginning on GDs 34, 45, 55, or 65 ± 1 of the normal 114‐day gestational period. These timepoints were chosen to capture a period beginning prior to the onset of fetal thyroid function in the pig (Brzezińska‐Slebodzińska and Slebodziński 2004) and ending at the start of the late gestation period. Within each timepoint, gilts were randomly assigned to one of two treatment groups, with *n* = 3 gilts per timepoint receiving a 5 mg/kg daily dosage of methimazole (MMI) (Sigma‐Aldrich, St. Louis, MO, USA) dissolved in 50% corn syrup/50% water and administered in 200 g supplemental feed, and *n* = 3 gilts per timepoint receiving an equivalent sham control (CON). The 5 mg/kg dosage of MMI was chosen based on prior research showing that a 21‐day treatment period at this dose was sufficient to induce severe porcine fetal hypothyroidism (Ison et al. 2023). Throughout the study, gilts were weighed twice weekly, and dosages adjusted accordingly.

### 2.2 Sample Collection

After 21 days of treatment and at GDs 55, 66, 76, and 86 ± 1, respectively, the gilts were humanely euthanized via captive bolt followed by exsanguination. Following the euthanasia of the dam, a midline ventral incision was made to allow for the removal of the gravid uterus, which was extracted and linearized to allow for the sequential removal of the fetuses. Fetuses were removed from each horn starting at the uterotubal junction and working towards the cervix, with fetal position, sex, and viability recorded using previously described methods (Mulligan et al. 2022). Immediately following the removal from the uterus, fetal blood samples were collected from all viable fetuses (*n* = 335) via the axillary artery, and the resultant serum stored at −20°C for later analyses. Within each litter, the most centrally located viable male and female fetus from each uterine horn was selected for extensive sampling (*n* = 96 select fetuses). Each fetus was subsequently weighed and dissected, and an apical portion of a fetal liver (LVR) lobe and a sample of fetal kidney (KID) collected after first weighing the whole organs. Tissues were snap‐frozen in liquid nitrogen, followed by storage at −80°C for later use in gene expression analyses. All animal procedures were carried out in compliance with Purdue University's animal care policies and approved by the Institutional Animal Care and Use Committee (IACUC Protocol #0123002344).

### 2.3 Fetal Thyroid Histology

Fetal thyroids (ROID) were isolated from all *n* = 96 select fetuses, weighed, and fixed in 10% neutral buffered formalin for histological assessment. Tissues were processed and paraffin embedded by the Purdue Histology Research Laboratory. Then, 8 μm thick sections were cut on an HM 325 rotary microtome and allowed to dry overnight. Slides were subsequently baked at 60°C for 15 min and deparaffinized using xylene, followed by rehydration with a series of graded ethanol concentrations. Resultant slides were stained with hematoxylin and eosin (Thermo Fisher, Waltham, MA, USA) and cover‐slipped with xylene mounting medium (Thermo Fisher), followed by assessment and imaging of representative view fields on a standard light microscope using a 40× objective and 1080P camera.

### 2.4 Circulating Thyroid Hormone Measurements

For measurement of total circulating thyroid hormone levels, commercial chemiluminescent immunoassay kits for T3 (SKU: 07M175‐CF) and T4 (SKU: 07M275‐CF), which have previously been shown to work in swine (Fazioli et al. 2024), were utilized according to the manufacturer's instructions (MP Biomedicals, Solon, OH, USA). Briefly, 50 and 25 μL serum aliquots from select fetuses were assayed in duplicate for T3 and T4, respectively, with an independent pool of fetal serum and an independent pool of postnatal serum serving as inter‐plate controls. Where necessary due to low sample volumes obtained at earlier GDs, samples from non‐select fetuses were substituted for use in either assay to allow for measurement of *n* = 12 fetuses per treatment group at each GD. Relative luminescent units in each well were measured on a Spark 10 M spectrophotometer (Tecan Life Sciences, Männedorf, Switzerland), and resultant thyroid hormone levels calculated relative to a standard curve. The average inter‐ and intra‐assay variances for T3 were 8.42% and 6.67%, respectively, with the average inter‐ and intra‐assay variances for T4 being 9.27% and 5.49%, respectively.

### 2.5 RNA Isolation and Reverse Transcription

Flash‐frozen samples of the fetal LVR and KID were ground to a fine powder under liquid nitrogen in a pre‐chilled mortar and pestle. RNA was extracted from a ~100 mg aliquot using a double‐precipitation protocol with TRIzol reagent (Thermo Fisher), followed by DNase treatment using the Turbo DNA‐free kit (Thermo Fisher) with the addition of 20 IU of RNaseOUT in each reaction (Thermo Fisher). Concentration and purity of the resultant RNA were determined spectrophotometrically on a Nanodrop ND1000 (Thermo Fisher), and integrity was assessed via a 1.2% (w/v) denaturing agarose gel. A total of 2 μg of RNA from each sample was reverse transcribed using the High‐Capacity cDNA Reverse Transcription kit (Applied Biosystems, Foster City, CA, USA) with the addition of 20 IU RNaseOUT included in each reaction. Resultant cDNA libraries were diluted to 10 ng/μL and stored at −20°C for later use. Due to sample loss of one fetal LVR from each of the CON groups at GDs 55 and 76, the final LVR sample number for these respective groups was *n* = 11, rather than the *n* = 12 for all other groups.

### 2.6 Gene Expression Analysis

Conventional PCR primers were designed using Primer‐BLAST to pick up all known and predicted transcript variants for thyroid hormone receptor alpha (*THRA*) and thyroid hormone receptor beta (*THRB*) present in the Sscrofa11.1 genome assembly (Table 1). To assess the presence or absence of expression of these genes in the fetal LVR and KID, cDNA samples from six CON fetuses per each GD were pooled for PCR. Reactions were run in a PTC‐200 thermal cycler (MJ Research, Quebec, Canada), with 10 μL Phusion Flash High‐Fidelity PCR Master Mix (Thermo Fisher), 20 ng cDNA, and 0.5 μM of each forward and reverse primer used in a total reaction volume of 20 μL. A total of 30 cycles were run with a 15 s extension time, and denaturation and annealing steps programmed according to the manufacturer's directions. Following completion of the reaction, a 10 μL aliquot of product was run on a 1% agarose gel. Bands were visualized using SYBRSafe (Invitrogen, Waltham, MA, USA), with amplicon length assessed relative to a 1 kb DNA ladder (Thermo Fisher).

**TABLE 1:** Porcine specific primer sequences used for RT‐qPCR and PCR.

	Gene ID	Symbol	Forward primer	Reverse primer	Amplicon length	Annealing temp	Target sequence/reference
Reference (RT‐qPCR)	414396	*ACTB*	5′‐CCAGCACGATGAAGATCAAG‐3′	5′‐AGTCCGCCTAGAAGCATTTG‐3′	171	61	Ison et al. (2023)
	396989	*RPL19*	5′‐AACTCCCGTCAGCAGATCC‐3′	5′‐AGTACCCTTCCGCTTACCG‐3′	147	61	Pasternak et al. (2015)
	780440	*YWHAZ*	5′‐TGATGATAAGAAAGGGATTGTGG‐3′	5′GTTCAGCAATGGCTTCATCA‐3′	203	60	Pasternak et al. (2020a)
	100628048	*STX5*	5′‐TGCAGAGTCGTCAGAATGGA‐3′	5′‐CCAGGATTGTCAGCTTCTCC‐3′	144	61	Pasternak et al. (2020b)
GOI (RT‐qPCR)	397491	*IGF1*	5′‐TTATTTCAACAAGCCCACAGG‐3′	5′‐CTCCAGCCTCCTCAGATCAC‐3′	106	58	NM_214256.1
	396916	*IGF2*	5′‐ACACCCTCCAGTTTGTCTGC‐3′	5′‐GGTATCTGGGGAAGTTGTCC‐3′	210	60	NM_213883.2
	397270	*IGFBP1*	5′‐CACAGCAAACAGTGCGAGA‐3′	5′‐GTGGAGCCCAGGATCTTCTT‐3′	92	61	NM_001195105.1
	397064	*IGFBP2*	5′‐GAGAAAGTCACGGAGCAGCA‐3′	5′‐TTGTCACAGTTGGGGATGTG‐3′	212	63	NM_214003.1
	448812	*IGFBP3*	5′‐CGGACACCCAGAACTTCTCC‐3′	5′‐TACTTATCCACGCACCAGCA‐3′	220	60	NM_001005156.1
	100144490	*IGFBP4*	5′‐GAGGAGCTGGTGCGAGAG‐3′	5′‐CACCCTCGTCCTTGTCAGA‐3′	232	58	NM_001123129.1
	397182	*IGFBP5*	5′‐GGTTTGCCTCAACGAAAAGA‐3′	5′‐ACGAACTTGGACTGGGTCAG‐3′	204	60	NM_214099.2
	100101923	*IGFBP6*	5′‐GGAGAGTAAGCCCCAAGCAG‐3′	5′‐TTAGGCACGTAGAGGGTGTG‐3′	213	63	NM_001100190.1
	100302573	*IGFBP7*	5′‐AACTCCCAAGGACAGGCTTC‐3′	5′‐CAGCTCAGCACCTTCACCTT‐3′	93	61	NM_001163801.1
GOI (PCR)	397387	*THRA*	5′‐CCTGGACAAAGACGAGCAGT‐3′	5′‐CGGAATGTTGTGTTTGCGGT‐3′	946	65	NM_214190.1
	396776	*THRB*	5′‐TGCCTTCAGTGAGCCCAAAA‐3′	5′‐GAAACGGAGAGGGTCAGTGG‐3′	508	65	XM_013981418.2

For RT‐qPCR, primer sequences for reference genes were extracted from prior literature, while Current RefSeq mRNA sequences were used to design primers for genes of interest (GOI) (Table 1). Primers were designed to span exon‐exon junctions identified using the BLAST‐like alignment tool (BLAT) relative to the Sscrofa11.1 genome assembly. Target specificity and coverage of all known and predicted porcine transcript variants for each target was confirmed using the Basic Local Alignment Search Tool (BLAST). Additionally, all designed primer pairs were determined to have a ΔG for the most favorable hetero‐ and homodimers of > −7.5 kcal/mol. Before use in RT‐qPCR assays, primers were first validated to confirm both the production of a single amplicon product, as well as the efficiency of the primer pair, with all primers having an efficiency between 93% and 109% as determined by a 5‐point serial dilution. Validated primers were subsequently used to assess the relative abundance of each reference gene and GOI in the fetal LVR and KID cDNA samples, with 20 ng cDNA used per reaction and reactions run in a 96‐well plate on a CFX Connect qPCR system (Bio‐Rad, Hercules, CA, USA) with SsoAdvanced Universal SYBR Green Supermix (Bio‐Rad). Primers were used at a concentration of 0.33 μM, and all samples were assayed in duplicate, with the coefficient of variance between all duplicate samples equal to or < 1%.

### 2.7 Western Ligand Blotting

Serum IGFBP levels were assessed in a subset of fetuses using Western Ligand Blotting as previously described (Houle et al. 1997; Wirthgen et al. 2016). A total of *n* = 95 fetuses were selected from the overall population of *n* = 335, with a sample size of *n* = 12 for all groups except for the MMI group at GD 55, which had a final sample size of *n* = 11. Prior to blotting, recombinant human IGF1 protein (R&D Systems, Minneapolis, MN, USA) was biotinylated using a 50‐fold molar excess of the EZ‐Link Sulfo‐NHS Biotin reagent (Thermo Fisher), with unbound biotin removed using a 3 kilodalton molecular weight cutoff centrifugal filter unit (Amicon, MilliporeSigma, Burlington, MA, USA). Subsequently, 1.5 μL of serum sample was diluted in SDS loading buffer, denatured at 95°C for 5 min under non‐reducing conditions, and run down a 12% SDS‐PAGE gel. Gels were transferred onto 0.45 μm nitrocellulose membranes using a semi‐dry transfer system, and resultant blots were blocked for 1 h at room temperature in 2% skim milk diluted in tris‐buffered saline (TBS). Blots were subsequently incubated for 2 h at room temperature in 200 ng/mL biotinylated IGF1 diluted in TBS with the addition of 0.1% Tween 20 (TBST). After the IGF1 incubation, blots were incubated in a 1:200 dilution of streptavidin‐HRP (R&D Systems) in TBST for 1 h at room temperature. Detection was performed using enhanced chemiluminescence (Bio‐Rad), and blots were imaged with a ChemiDoc imaging system (Bio‐Rad), with images of various exposure times taken to allow for quantification of a wide range of band intensities.

In addition to samples of interest, each blot contained a lane of pooled recombinant human protein standards representing IGFBPs ‐1, ‐2, ‐3, and ‐4 (R&D Systems), which were used as reference points for identification of bands of interest, as well as a lane of pooled fetal serum which was used to calculate the relative density of each band of interest for analysis. All densitometric analyses were performed in Image Lab version 6.1.0. Briefly, bands were manually identified, and background subtraction was performed consistently within each blot. The relative density of each band was then calculated by dividing the background‐adjusted volume of the band by the volume of the equivalent band in the fetal serum pool that was run on each gel.

### 2.8 Statistical Analyses

All statistical analyses and data visualization were performed in R version 4.3.1 (R Core Team 2023), with figures generated using ggplot2 (Wickham 2016). Fetal phenotype data and Western Ligand Blotting data was analyzed using a linear model including treatment group, GD, and the interaction effect. Data were first visually assessed for normality and log transformed prior to analysis to improve normality as needed, but all results were presented on the original scale. Pairwise contrasts were performed using the emmeans package (Lenth 2024) with a Šidák correction for multiple comparisons to first establish the ontogenic trajectory of fetal phenotype and sera IGFBP levels throughout gestation in the CON group, and then to assess the differences resulting from MMI treatment within each timepoint. Data on circulating thyroid hormone levels was analyzed nonparametrically with a Kruskal–Wallis test, followed by a pairwise Wilcoxon signed‐rank test with a Bonferroni correction to allow for comparisons between both the CON and MMI groups within each GD, as well as within the CON group over time.

For analysis of gene expression using RT‐qPCR, the stability of presumptive reference genes was initially assessed using both parametric and non‐parametric approaches. As with prior studies of porcine fetal ontogeny, the stability of these canonical reference genes could not be demonstrated over time (Figure S1), and thus normalization was accomplished using the previously demonstrated residual approach (Novak et al. 2012; Vallet et al. 2010). First, the Cts for each presumptive reference gene were fit to a linear model including GD, treatment group, and the interaction. The residuals from the resultant models were found to be highly correlated, suggesting they are indicative of technical error (Figure S2C,F). Following this validation, the geometric means (GEO) of the three most stable reference genes in each tissue (*ACTB*, *YWHAZ*, and *RPL19* in the LVR; *ACTB*, *YWHAZ*, and *STX5* in the KID) were then fit to a linear model and the residuals extracted and used to calculate normalized Ct (ΔCt) values for each GOI. The issue of ontogenic changes in reference genes and the stability of residual correction factors resulting from utilizing this method are shown in Figure S2A–E. Following normalization, the ΔCts for each GOI were fit to a linear model including GD, treatment group, and the interaction, either with or without gilt as a random effect. Assessment of the Akaike and Bayesian information criterion (AIC and BIC) between models indicated that the inclusion of the random effect term decreased model fit, and it was subsequently removed prior to further analyses. Similar to the assessment of the phenotype data, pairwise contrasts were performed using the emmeans package (Lenth 2024) with a Šidák correction for multiple comparisons to first establish the ontogenic trajectory of the GOIs over time within the CON group, and then to assess expression differences resulting from MMI treatment within each timepoint. The model emmeans and 95% confidence intervals for each gene were converted to fold changes using the 2^−ΔΔCt^ method to allow for data visualization. Finally, the relationship between the IGFBPs within tissues from CON fetuses was assessed via Pearson's correlation coefficient of the normalized C_t_ values calculated above, with results visualized using a custom script based on the grid package in base R. For all analyses, the threshold for statistical significance was set at *p* < 0.05, with results between 0.05 < *p* < 0.10 reported as trends.

## 3 Results

### 3.1 Fetal Phenotypes

Fetal phenotype data for the select fetal subset is shown in Table 2. Among the CON groups, fetal body weight, absolute KID weight (of both KIDs combined), and absolute ROID weight were significantly increased across all timepoints as gestation progressed (*p* < 0.001 for all comparisons except for GD 66 vs. GD 76 KID weight, where *p* = 0.005, and GD 66 vs. GD 76 ROID weight, where *p* = 0.014). Similarly, absolute fetal LVR weight was significantly increased at GD 86 relative to all other GDs (*p* < 0.001 for all comparisons), and also at GD 76 relative to GD 55 (*p* < 0.001), with an additional trend observed towards increased weight at GD 76 relative to GD 66 (*p* = 0.095). Opposite of that observed for absolute organ weights, the relative weights of the fetal LVR and KID decreased as time progressed, with both relative LVR and KID weight significantly decreased at GDs 76 and 86 relative to GDs 55 and 66 (*p* < 0.001 for all comparison), and also decreased at GD 66 relative to GD 55 (*p* < 0.001 for the LVR and *p* = 0.002 for the KID). In contrast, relative ROID weight was not altered throughout gestation within the CON group.

**TABLE 2:** Body weight, absolute liver (LVR), kidney (KID), and thyroid (ROID) weight, and relative LVR, KID, and ROID weight of select fetuses (*n* = 11–12/group) at various gestational days (GDs) following 21 days of maternal CON or MMI treatment.

	GD	55		66		76		86		*p*		
	Group	CON	MMI	CON	MMI	CON	MMI	CON	MMI	Group	GD	Interaction
Absolute weight (g)	Body	85.092^a^	78.608	174.291^b^	212.292	304.161^c^	336.818	585.041^d^	532.386	0.362	< 0.001	0.007
		(77.281–93.693)	(71.392–86.553)	(158.292–191.907)	(192.805–233.748)	(276.241–334.902)	(305.900–370.860)	(531.338–644.172)	(483.517–586.195)			
	LVR	5.171^a^	5.183	6.970^ab^	8.927*	8.966^b^	11.338**	15.936^c^	15.305	0.049	< 0.001	0.029
		(4.024–6.318)	(4.036–6.330)	(5.824–8.117)	(7.781–10.074)	(7.819–10.112)	(10.192–12.485)	(14.789–17.082)	(14.108–16.503)			
	KID	1.463^a^	1.336	2.398^b^	2.821	3.236^c^	3.919	5.685^d^	5.716	0.223	< 0.001	0.078
		(1.295–1.653)	(1.182–1.509)	(2.122–2.709)	(2.496–3.187)	(2.864–3.656)	(3.469–4.428)	(5.032–6.424)	(5.031–6.493)			
	ROID	0.020^a^	0.033**	0.039^b^	0.242***	0.061^c^	0.359***	0.114^d^	0.412***	< 0.001	< 0.001	< 0.001
		(0.017–0.025)	(0.027–0.040)	(0.032–0.048)	(0.199–0.295)	(0.050–0.074)	(0.296–0.436)	(0.094–0.139)	(0.336–0.505)			
Relative weight (% body weight)	LVR	5.979^a^	6.578**	3.908^b^	4.190	2.919^c^	3.382*	2.647^c^	2.843	< 0.001	< 0.001	0.389
		(5.730–6.228)	(6.326–6.825)	(3.659–4.158)	(3.941–4.439)	(2.670–3.168)	(3.132–3.631)	(2.398–2.896)	(2.582–3.103)			
	KID	1.719^a^	1.699	1.378^b^	1.329	1.064^c^	1.164	0.972^c^	1.071	0.187	< 0.001	0.268
		(1.583–1.866)	(1.565–1.845)	(1.267–1.494)	(1.224–1.443)	(0.980–1.155)	(1.072–1.263)	(0.895–1.055)	(0.983–1.167)			
	ROID	0.024	0.041***	0.023	0.114***	0.020	0.107***	0.020	0.077***	< 0.001	< 0.001	< 0.001
		(0.020–0.029)	(0.035–0.049)	(0.019–0.027)	(0.096–0.136)	(0.017–0.024)	(0.090–0.127)	(0.016–0.023)	(0.064–0.093)			

*Note:* ^a,b,c,d^Significant differences (*p* < 0.05) between the CON groups over time are represented with unique letter superscripts. *Significant differences between the CON and MMI group within each timepoint are denoted as *p < 0.05, **p < 0.01, and ***p < 0.001. Data are presented as the estimated marginal means with 95% confidence intervals, as extracted from a linear model including treatment group, gestational day (GD), and the interaction.

Within each timepoint, treatment with MMI had temporally specific impacts on absolute and relative LVR weight, with no impact on absolute or relative KID weight. Absolute LVR weight was increased in MMI fetuses relative to age‐matched CONs at GDs 66 and 76 (*p* = 0.019 and *p* = 0.005), while relative LVR weight was increased in the MMI fetuses at GDs 55 and 76 (*p* = 0.001 and *p* = 0.011). As expected, the induction of hypothyroidism in MMI fetuses was marked by increases in both absolute and relative ROID weight at all studied timepoints (*p* < 0.001 for all comparisons except for GD 55 vs. GD 66 absolute ROID weight, where *p* = 0.001). A significant time by treatment group interaction was observed for body weight (*p* = 0.007), absolute LVR weight (*p* = 0.029), absolute ROID weight (*p* < 0.001), and relative ROID weight (*p* < 0.001), with a trend towards interaction significance for absolute KID weight (*p* = 0.078).

### 3.2 Fetal Thyroid Histology

**FIGURE 1:**
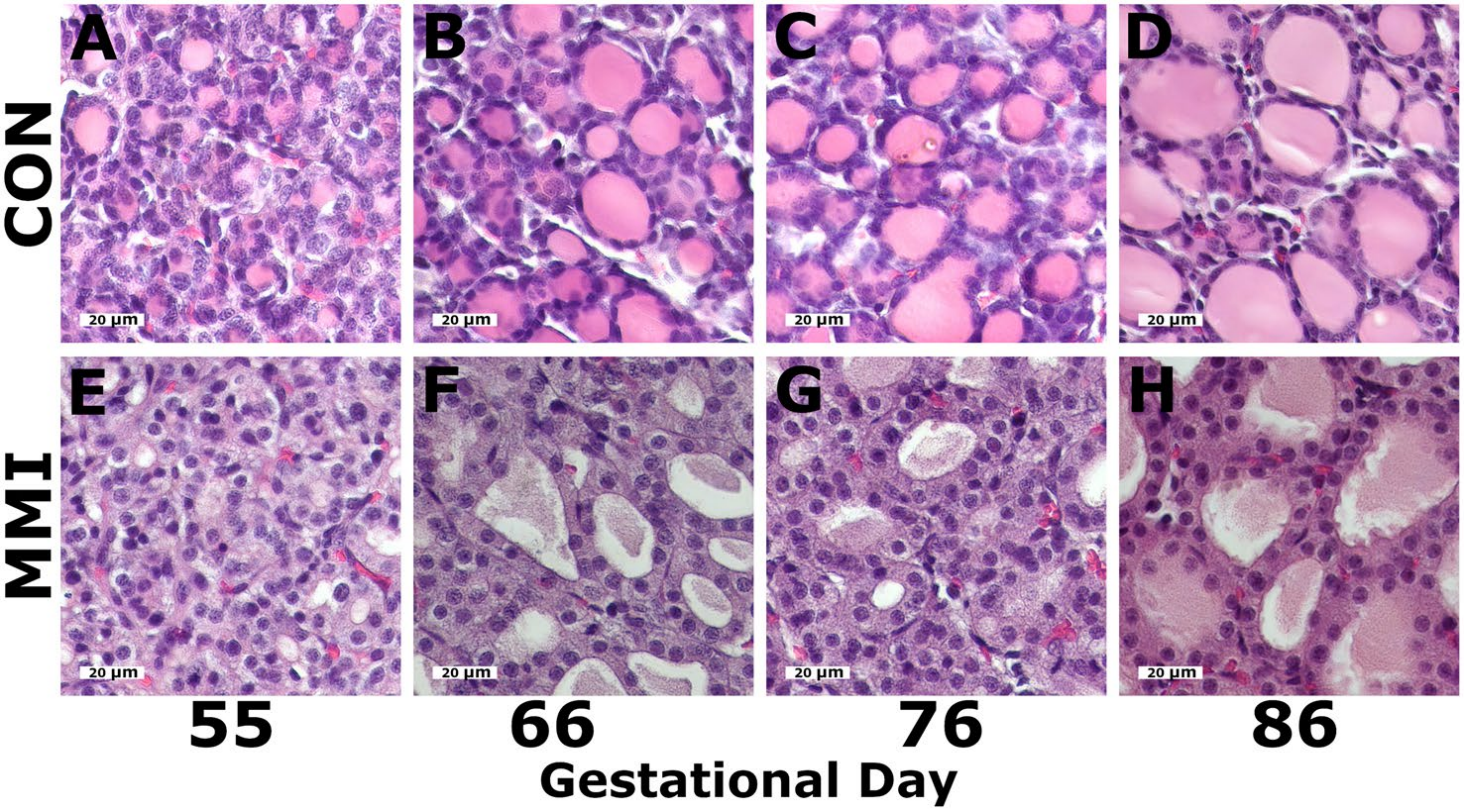
Fetal thyroid histology. Representative images of fetal thyroid histology following 21 days of maternal CON or MMI treatment, with CON thyroids at gestational days (A) 55, (B) 66, (C) 76, and (D) 86 exhibiting normal follicular shape and staining of the eosinophilic colloid, and MMI thyroids at gestational days (E) 55, (F) 66, (G) 76, and (H) 86 exhibiting a loss of follicular structure and a lack of staining in the eosinophilic colloid.

Representative images of fetal ROID histology from the CON and MMI groups at all four GDs are shown in Figure 1, with additional examples shown in Figure S3. All CON fetuses exhibited normal, healthy follicular morphology (Figure 1A–D), as evidenced by largely spherical follicles lined by a single layer of cuboidal epithelial cells and containing colloid with a strong staining affinity for eosin. Relative to the age‐matched CONs, the MMI ROIDs at all four timepoints exhibited a decrease in colloid staining intensity indicative of hypothyroidism (Figure 1E–H). At GDs 66, 76, and 86, a marked loss of the normal spherical shape of the follicles could also be observed in the MMI fetuses, with MMI follicles at GD 55 appearing largely underdeveloped. No marked alterations were noted in the morphology of the follicular epithelium at any timepoint.

### 3.3 Fetal Circulating Thyroid Hormone Levels

**FIGURE 2:**
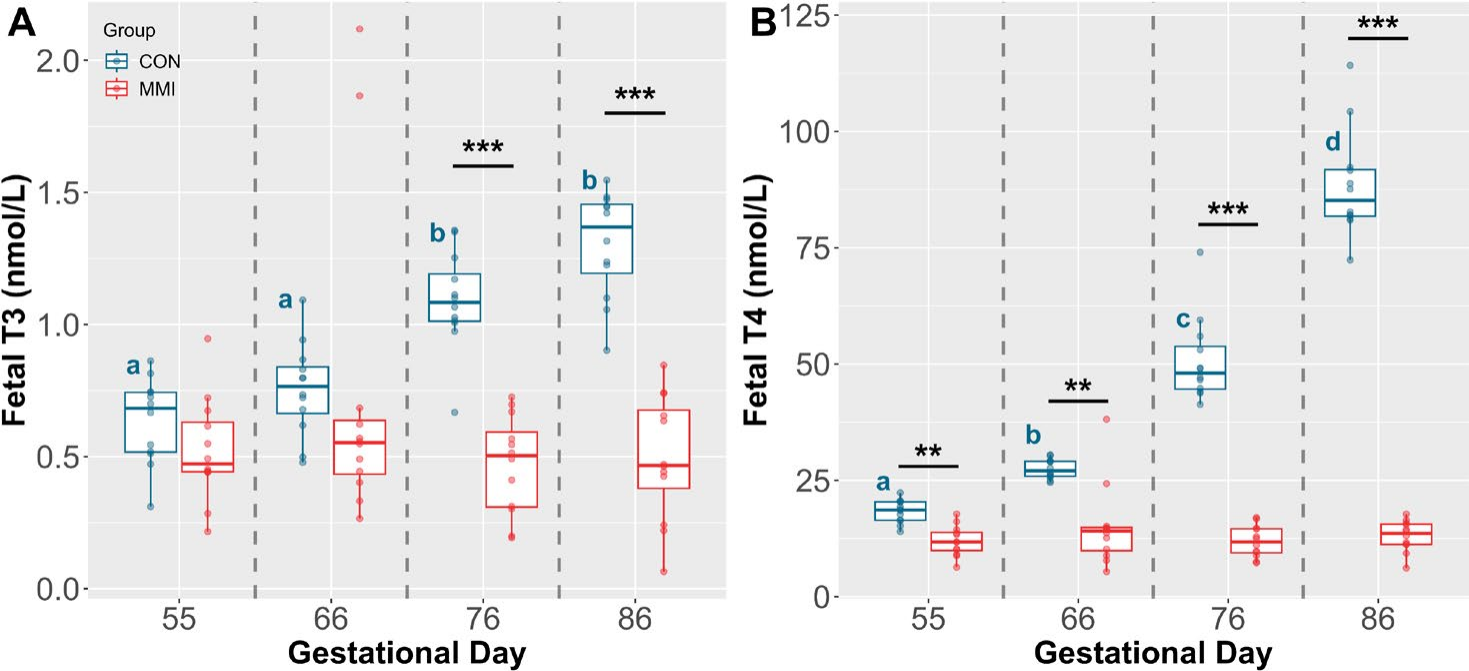
Fetal circulating thyroid hormone levels. Levels of fetal (*n* = 12/group) circulating (A) T3 and (B) T4 at various gestational timepoints following 21 days of maternal CON or MMI treatment, with concentrations presented in standard SI units of nmol/L. Unique letter superscripts denote statistically significant (*p* < 0.05) changes in circulating concentration in the CON group over time, with statistical differences between the CON and MMI groups within each timepoint denoted as *p < 0.05, **p < 0.01, and ***p < 0.001.

Among the CON groups, ontogenic changes in both T3 (Figure 2A) and T4 (Figure 2B) were observed over time, with T3 being significantly increased at GD 76 relative to GDs 55 and 66 (*p* < 0.001 and *p* = 0.010, respectively), and also significantly increased at GD 86 relative to GDs 55 and 66 (*p* < 0.001 for both comparisons). In contrast, no significant increases in T3 were detected in the CON groups between GDs 55 and 66, or between GDs 76 and 86. Changes in T4 levels among the CON groups were statistically significant for all comparisons, with there being significant increases in circulating T4 between each assessed timepoint (*p* < 0.001 for all comparisons). The median levels of circulating thyroid hormones in the CON groups at all four timepoints were 0.683, 0.766, 1.084, and 1.369 nmol/L for T3, and 18.628, 27.037, 48.066, and 85.141 nmol/L for T4.

Within each timepoint, circulating fetal T3 was significantly reduced in the MMI group relative to CON at GDs 76 and 86 (*p* < 0.001 for both comparisons), but not at GDs 55 or 66. In contrast, fetal sera T4 was significantly reduced in the MMI groups relative to CON at all studied gestational timepoints (*p* = 0.001, *p* = 0.006, *p* < 0.001, and *p* < 0.001, respectively, for GDs 55, 66, 76, and 86). Interestingly, when comparing the residual levels of thyroid hormones detected in the MMI groups, no significant differences in either T3 or T4 levels were noted across the four GDs. As such, the median levels of circulating thyroid hormones in the MMI groups at all four timepoints were 0.472, 0.553, 0.503, and 0.467 nmol/L for T3, and 11.738, 14.067, 11.754, and 13.587 nmol/L for T4.

### 3.4 *THRA* and *THRB* Gene Expression

**FIGURE 3:**
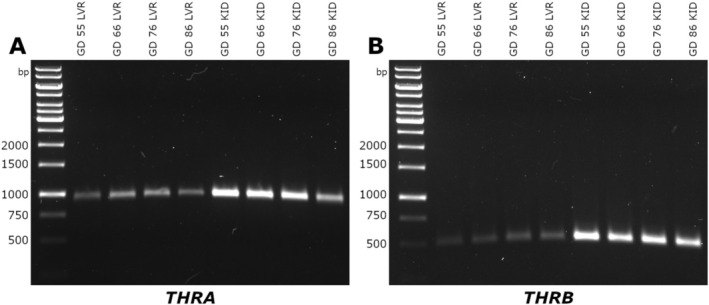
Thyroid hormone receptor PCR products in fetal liver (LVR) LVR and kidney (KID). Agarose gel of PCR products for (A) *THRA* and (B) *THRB* in CON fetal LVR and KID cDNA pools at gestational days (GDs) 55, 66, 76, and 86, with a DNA ladder shown in the left‐most lane of each gel for reference.

Bands representing PCR products for *THRA* and *THRB* in pooled CON cDNA samples of fetal LVR and KID are shown in Figure 3. The *THRA* product (Figure 3A) was detected at the expected base pair length of 946 in both the LVR and KID at all four studied time points, with a more robust band noted in the KID. Similarly, the product for *THRB* (Figure 3B) was detected at the expected length of 508 base pairs in all pooled samples, with a more robust band also noted in the KID.

### 3.5 *IGF1* and *IGF2* Gene Expression

**FIGURE 4:**
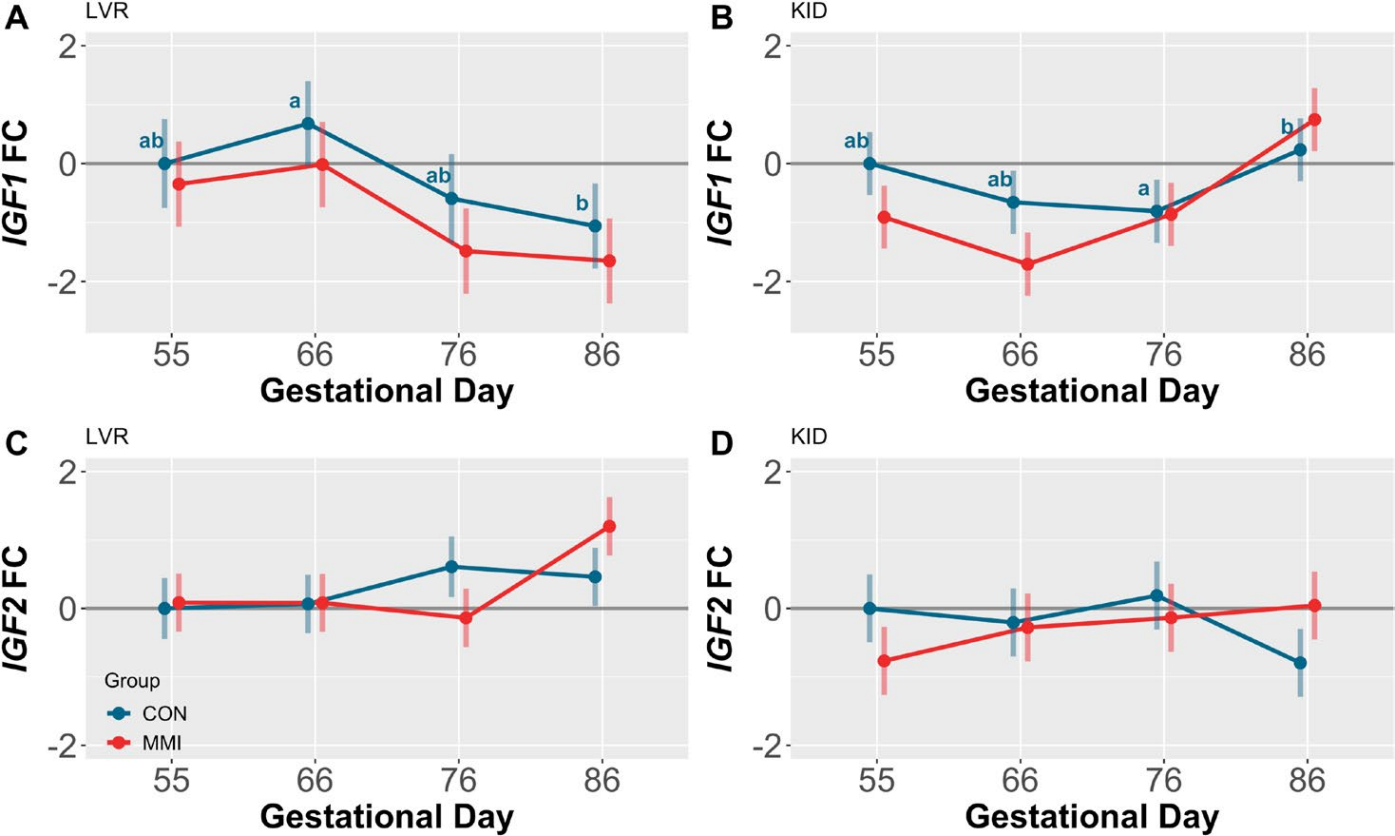
Fetal liver (LVR) and kidney (KID) gene expression of IGF ligands. Expression of (A, B) *IGF1* and (C, D) *IGF2* in fetal (A, C) LVR and (B, D) KID tissue derived from fetuses (*n* = 11–12/group) at various gestational timepoints following 21 days of maternal CON or MMI treatment. All fold changes (FC) were calculated relative to the CON group at gestational day 55, with unique letter superscripts denoting statistically significant (*p* < 0.05) changes in gene expression in the CON group over time. Within each timepoint, no significant differences were observed in expression of either gene in either tissue as a result of MMI treatment. Data are presented as the estimated marginal means with 95% confidence intervals, as extracted from a linear model including gestational day, treatment group, and the interaction.

Among the CON groups, ontogenic changes in expression of *IGF1* were observed in both the fetal LVR (Figure 4A) and KID (Figure 4B), with hepatic expression of *IGF1* decreasing significantly between GDs 66 to 86 (*p* = 0.007) and renal expression increasing between GDs 76 to 86 (*p* = 0.045). In contrast, *IGF2* exhibited no ontogenic changes over time in either the LVR (Figure 4C) or KID (Figure 4D). Within each timepoint, treatment with MMI had no impact on expression of either *IGF1* or *IGF2* in either tissue, with only a trend observed towards downregulation of *IGF1* in the LVR of MMI fetuses at GD 76 (*p* = 0.093). However, a significant time by treatment interaction was observed for renal *IGF1* expression (*p* = 0.014), as well as hepatic *IGF2* (*p* = 0.011) and renal *IGF2* (*p* = 0.015) expression.

### 3.6 Hepatic IGFBP Gene Expression

**FIGURE 5:**
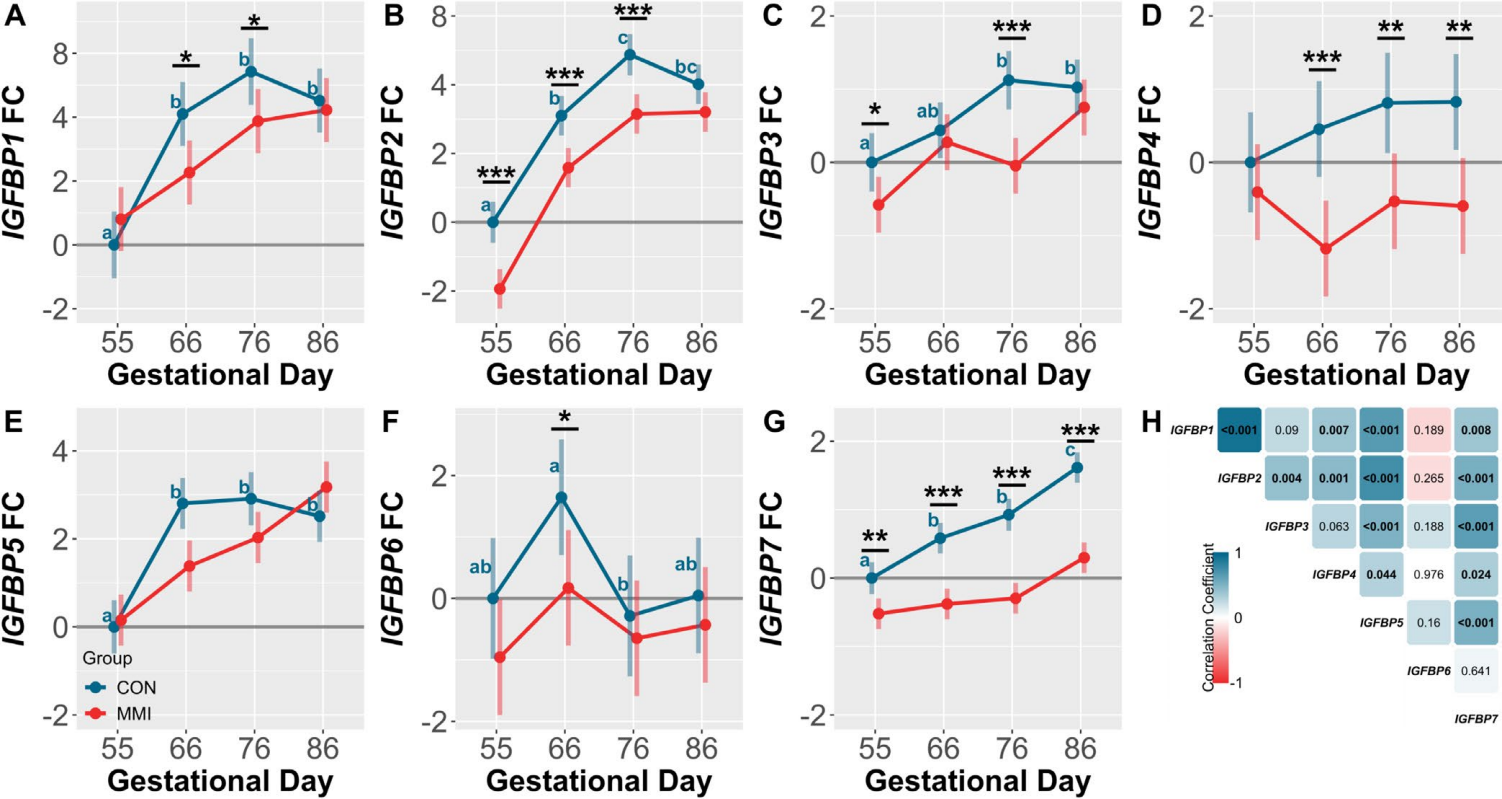
Fetal gene expression of hepatic IGFBPs. Expression of (A) *IGFBP1*, (B) *IGFBP2*, (C) *IGFBP3*, (D) *IGFBP4*, (E) *IGFBP5*, (F) *IGFBP6*, and (G) *IGFBP7* in liver tissue derived from fetuses (*n* = 11–12/group) at various gestational timepoints following 21 days of maternal CON or MMI treatment. All fold changes (FC) were calculated relative to the CON group at gestational Day 55, with unique letter superscripts denoting statistically significant (*p* < 0.05) changes in gene expression in the CON group over time, and statistical differences between the CON and MMI group within each timepoint denoted as *p < 0.05, **p < 0.01, and ***p < 0.001. Data are presented as the estimated marginal means with 95% confidence intervals, as extracted from a linear model including gestational day, treatment group, and the interaction. (H) Correlation matrix showing the relationship between normalized hepatic IGFBP Ct values in CON fetuses, with color indicating the Pearson correlation coefficient, and the associated *p*‐value inscribed within each cell and represented in bold text if significant.

Among CON fetuses, ontogenic changes in the expression of the hepatic IGFBPs were observed for all assessed genes except for *IGFBP4* (Figure 5D). Both *IGFBP1* (Figure 5A) and *IGFBP5* (Figure 5E) were significantly increased at GDs 66, 76, and 86 relative to GD 55 (*p* < 0.001 for all comparisons), with *IGFBP3* (Figure 5C) significantly increased at GDs 76 and 86 relative to GD 55 (*p* < 0.001 and *p* = 0.002, respectively). Additionally, a trend was observed towards upregulation of *IGFBP3* between GDs 66 to 76 (*p* = 0.088). Expression of *IGFBP2* (Figure 5B) was significantly increased at all timepoints relative to GD 55 (*p* < 0.001 for all comparisons), with expression also significantly increased between GDs 66 and 76 (*p* < 0.001). Similarly, *IGFBP7* (Figure 5G) was significantly increased at all timepoints relative to GD 55 (*p* = 0.003, *p* < 0.001, and *p* < 0.001 for GDs 66, 76, and 86, respectively), with expression also higher at GD 86 relative to GDs 66 and 76 (*p* < 0.001 for both comparisons). Finally, *IGFBP6* (Figure 5F) decreased significantly in expression between GDs 66–76 only (*p* = 0.035). Moreover, many significant positive correlations were observed among the expression of the IGFBPs in CON fetuses (Figure 5H), with *IGFBP1* positively correlated with the expression of *IGFBP2*, ‐*4*, ‐*5*, and ‐*7* (*r* = 0.926, 0.391, 0.660, and 0.386, respectively). *IGFBP2* was additionally correlated with the expression of *IGFBP3*, ‐*4*, ‐*5*, and ‐*7* (*r* = 0.417, 0.456, 0.739, and 0.528, respectively), and *IGFBP3* with the expression of *IGFBP5* and ‐*7* (*r* = 0.508 and *r* = 0.637, respectively). Finally, the expression of *IGFBP4* was positively correlated with the expression of *IGFBP5* (*r* = 0.299), and both *IGFBP4* and ‐*5* expression positively correlated with the expression of *IGFBP7* (*r* = 0.333 and *r* = 0.523, respectively). In contrast, hepatic expression of *IGFBP6* was not correlated with the expression of any of the other IGFBPs.

With the exception of *IGFBP5*, all IGFBPs experienced at least one significant downregulation in the LVR as a result of MMI‐induced hypothyroidism. *IGFBP6* appeared marginally impacted, with only a significant downregulation in the MMI group observed at GD 66 relative to age‐matched CONs (*p* = 0.030). In contrast, *IGFBP1* was significantly downregulated in MMI fetuses at GDs 66 and 76 (*p* = 0.012 and *p* = 0.036, respectively), while *IGFBP3* was significantly downregulated at GDs 55 and 76 (*p* = 0.039 and *p* < 0.001, respectively). Both *IGFBP2* and ‐*4* were significantly downregulated at three of the four studied GDs, with *IGFBP2* downregulated at GDs 55, 66, and 76 (*p* < 0.001 for all comparisons), and *IGFBP4* downregulated at GDs 66, 76, and 86 (*p* < 0.001, *p* = 0.006, and *p* = 0.003, respectively). An additional trend towards downregulation of *IGFBP2* was also observed at GD 86 (*p* = 0.051). *IGFBP7* was uniquely downregulated at all studied gestational timepoints (*p* = 0.002, *p* < 0.001, *p* < 0.001, and *p* < 0.001, for GDs 55, 66, 76, and 86, respectively). In addition, a significant time by treatment interaction was observed for *IGFBP1*, ‐*5*, and ‐*7* (*p* = 0.043, *p* = 0.002, and *p* = 0.004, respectively), with a trend towards interaction significance for *IGFBP3* (*p* = 0.051), and no significant time by treatment interaction observed for *IGFBP2*, ‐*4*, or ‐*6*.

### 3.7 Renal IGFBP Gene Expression

**FIGURE 6:**
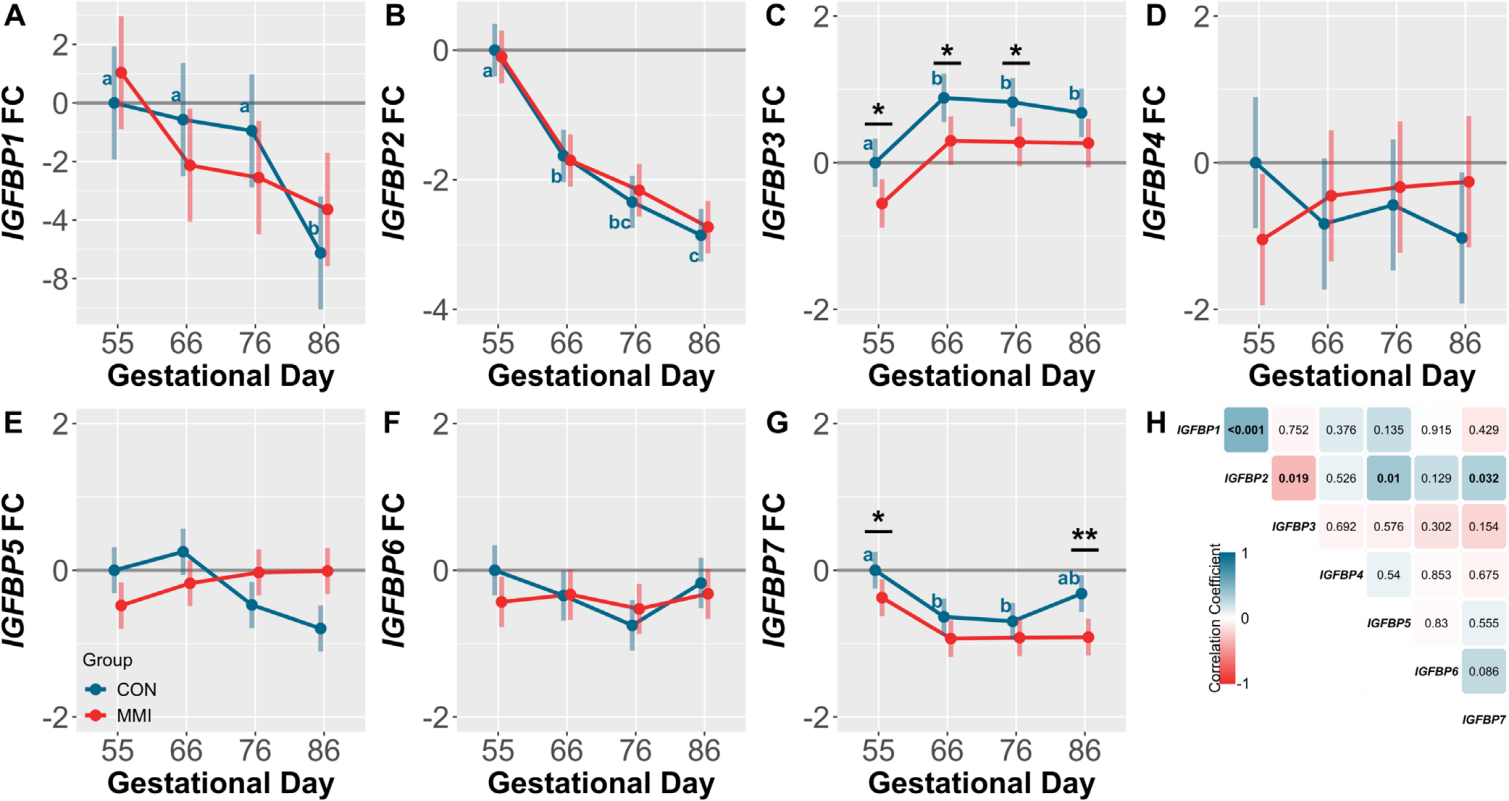
Fetal gene expression of renal IGFBPs. Expression of (A) *IGFBP1*, (B) *IGFBP2*, (C) *IGFBP3*, (D) *IGFBP4*, (E) *IGFBP5*, (F) *IGFBP6*, and (G) *IGFBP7* in kidney tissue derived from fetuses (*n* = 12/group) at various gestational timepoints following 21 days of maternal CON or MMI treatment. All fold changes (FC) were calculated relative to the CON group at gestational day 55, with unique letter superscripts denoting statistically significant (*p* < 0.05) changes in gene expression in the CON group over time, and statistical differences between the CON and MMI group within each timepoint denoted as *p < 0.05, **p < 0.01, and ***p < 0.001. Data are presented as the estimated marginal means with 95% confidence intervals, as extracted from a linear model including gestational day, treatment group, and the interaction. (H) Correlation matrix showing the relationship between normalized renal IGFBP Ct values in CON fetuses, with color indicating the Pearson correlation coefficient, and the associated *p*‐value inscribed within each cell and represented in bold text if significant.

Ontogenic changes in renal IGFBP expression were observed for *IGFBP1* (Figure 6A), ‐*2* (Figure 6B), ‐*3* (Figure 6C), and ‐*7* (Figure 6G). *IGFBP1* was significantly downregulated at GD 86 relative to all other GDs (*p* = 0.002, *p* = 0.008, and *p* = 0.019 for GDs 55, 66, and 76, respectively), with expression of *IGFBP3* increased at GDs 66, 76, and 86 relative to GD 55 (*p* = 0.002, *p* = 0.004, and *p* = 0.028, respectively). In contrast, *IGFBP2* expression was significantly decreased at all timepoints relative to GD 55 (*p* < 0.001 for all comparisons), with there also being a significant decrease in expression observed between GDs 66 and 86 (*p* < 0.001), and an additional trend towards downregulation noted between GDs 66 and 76 (*p* = 0.090). Expression of *IGFBP7* was significantly decreased at GDs 66 and 76 relative to GD 55 only (*p* = 0.004 and *p* = 0.001, respectively). No ontogenic changes in expression of *IGFBP4* (Figure 6D), ‐*5* (Figure 6E), or ‐*6* (Figure 6F) were observed. When assessing the relationships between renal IGFBP expression in CON fetuses (Figure 6H), only a small number of significant correlations were observed. All correlations involved *IGFBP2*, which was significantly positively correlated with expression of *IGFBP1*, ‐*5*, and ‐*7* (*r* = 0.502, 0.366, and 0.310, respectively), and significantly negatively correlated with expression of *IGFBP3* (*r* = −0.338).

In comparison to the LVR, minimal changes in IGFBP gene expression were observed in the KID as a result of MMI treatment, with significant dysregulations only observed for *IGFBP3* and ‐*7*. Renal *IGFBP3* expression was significantly downregulated in MMI fetuses at GDs 55, 66, and 76 relative to age‐matched CONs (*p* = 0.020, *p* = 0.015, and *p* = 0.023, respectively), with an additional trend observed towards downregulation at GD 86 (*p* = 0.082). In contrast, *IGFBP7* was significantly downregulated in the MMI fetuses at GDs 55 and 86 only (*p* = 0.040 and *p* = 0.001, respectively). No significant changes in the expression of renal *IGFBP1*, ‐*2*, ‐*4*, ‐*5*, or ‐*6* were observed in response to MMI treatment. Finally, the only renal IGFBP exhibiting a significant time by treatment interaction was *IGFBP5* (*p* < 0.001).

### 3.8 Sera IGFBP Levels

**FIGURE 7:**
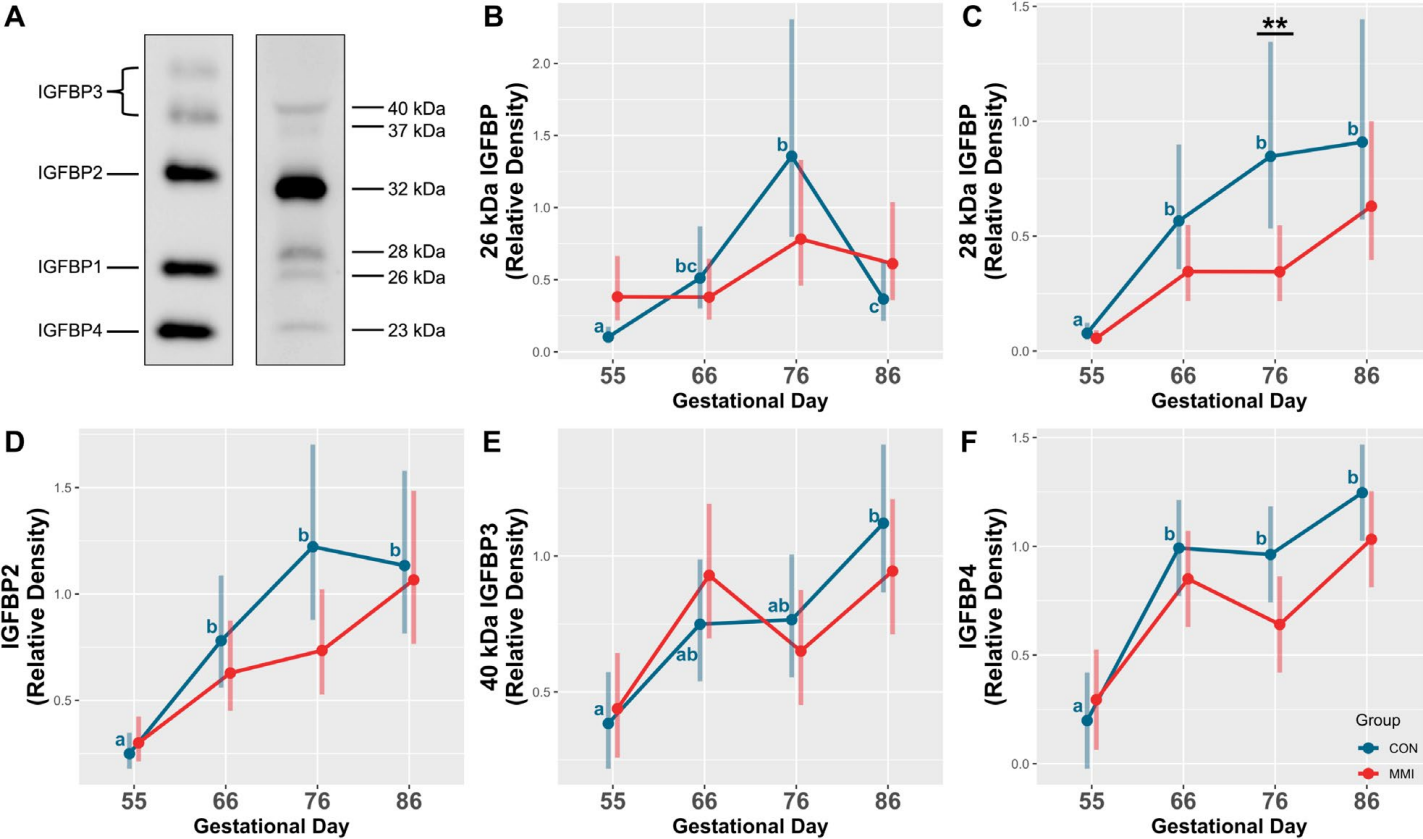
IGFBP levels in fetal serum. (A) Example Western Ligand Blot showing human recombinant protein standards in the left lane, with the right lane showing the six bands detected in fetal porcine serum. Relative density of (B) a 26 kDa IGFBP, (C) a 28 kDa IGFBP, (D) IGFBP2, (E) the 40 kDa IGFBP3, and (F) IGFBP4 in fetal porcine serum derived from fetuses (*n* = 11–12/group) at various gestational timepoints following 21 days of maternal CON or MMI treatment. Relative density was calculated relative to a consistent reference sample run on each gel, with unique letter superscripts denoting statistically significant (*p* < 0.05) changes in density in the CON group over time, and statistical differences between the CON and MMI group within each timepoint denoted as *p < 0.05, **p < 0.01, and ***p < 0.001. Data are presented as the estimated marginal means with 95% confidence intervals, as extracted from a linear model including gestational day, treatment group, and the interaction.

In fetal serum, six bands representing IGFBPs of various molecular weights were detected by Western Ligand Blotting (Figure 7A). Consistent with the human recombinant protein standard, IGFBP3 was detected as a doublet, with bands averaging 40 and 37 kDa under non‐reducing conditions. The 37 kDa band was very low in intensity and not able to be accurately quantified through densitometric analyses, and as a result, relative IGFBP3 levels were assessed through analysis of the 40 kDa band only. IGFBP2 and ‐4 were detected at approximate molecular weights of 32 and 23 kDa, respectively. Interestingly, two bands were detected around the IGFBP1 region, which were analyzed separately and labeled with their approximate molecular weights of 28 and 26 kDa, since the exact identity of these bands could not be elucidated with the present method.

Among CON animals, all five of the analyzed bands exhibited ontogenic increases in relative density throughout gestation. The 28 kDa IGFBP (Figure 7C), IGFBP2 (Figure 7D), and IGFBP4 (Figure 7F) were all increased in relative density at GDs 66, 76, and 86 relative to GD 55 (*p* < 0.001 for all comparisons). Similarly, the 40 kDa IGFBP3 band (Figure 7E) was increased in relative density at GD 86 relative to GD 55 (*p* < 0.001), with additional trends towards increased relative density at GDs 66 and 76 relative to GD 55 (*p* = 0.068 and *p* = 0.052, respectively). The 26 kDa IGFBP (Figure 7B) was increased in relative density at GDs 66, 76, and 86 relative to GD 55 (*p* < 0.001, *p* < 0.001, and *p* = 0.007, respectively), and also at GD 76 relative to GD 86 (*p* = 0.005), with an additional trend towards increased relative density at GD 76 relative to GD 66 (*p* = 0.067). Treatment with MMI had a minimal impact on sera IGFBP levels, with MMI fetuses having a significantly decreased relative density of the 28 kDa IGFBP band at GD 76 only (*p* = 0.008), and no significant impact of treatment observed for any of the other detected IGFBPs. Additionally, the only IGFBP exhibiting a significant time by treatment interaction was the 26 kDa IGFBP (*p* = 0.004).

## 4 Discussion

Thyroid hormones are critical metabolic regulators and are thought to play an important role in fetal development beginning early in gestation. In the human fetus, the thyroid is the first endocrine gland to develop (Kratzsch and Pulzer 2008), with the fetal pig following a similar developmental and functional trajectory (Brzezińska‐Slebodzińska and Slebodziński 2004). Aside from retrospective studies, there are two primary approaches for studying fetal hypothyroidism, being either surgical thyroidectomy (Spencer et al. 1989; Hopkins and Thorburn 1972; Young et al. 2023) or maternal administration of antithyroid drugs such as propylthiouracil or MMI (Elder et al. 2000; Näntö‐Salonen and Rosenfeld 1992). Our previous work has shown that MMI can cross the normally restrictive epitheliochorial porcine placenta, resulting in severe fetal hypothyroidism between GDs 85–106 (Ison et al. 2023). The present study expands on the utility of this model by showing that maternal MMI treatment can also induce porcine fetal hypothyroidism when administered earlier in gestation between GDs 34–86. Consistent with our prior work, successful induction of fetal hypothyroidism in the current study was supported by an increased ROID weight, the presence of abnormal, goitrous ROID histology, and concomitant decreases in sera T4 at all four GDs, with significant decreases of fetal T3 also observed at GDs 76 and 86.

The observed ontogenic increases in fetal T3 and T4 throughout gestation are consistent with prior studies across a multitude of species (Devaskar et al. 1986; Allen et al. 1995); however, the actual measured levels of circulating thyroid hormones in the present study are markedly higher than those previously reported for porcine fetuses of a similar gestational age (Brzezińska‐Slebodzińska and Slebodziński 2004). This discrepancy is consistent with recent publications in postnatal swine (Fazioli et al. 2024; Chapel et al. 2017) and has previously been hypothesized to be a byproduct of intensive selection for improved growth performance in contemporary swine (Pasternak et al. 2021). Additionally, among the MMI fetuses, it is interesting that similar thyroid hormone levels were measured across all four GDs, with neither hormone reaching undetectable levels. Similar to in humans (Roti et al. 1981), the porcine placenta possesses an enzymatic barrier that prevents transplacental transmission of bioactive maternal thyroid hormones (Krysin et al. 1997). The residual thyroid hormone levels in the MMI fetuses in the present study, however, may be indicative of a low level of thyroid hormone transplacental transfer in the MMI group. This hypothesis is supported by our prior work showing that placental metabolism of thyroid hormones may be altered in response to fetal endocrine status (Ison et al. 2023), as well as by an earlier study where low levels of thyroid hormones were still detected in fetal serum following thyroidectomy (Spencer et al. 1989). Alternatively, the observed residual levels of fetal thyroid hormones in the present study may be due to a persisting capacity of the fetal thyroid to produce thyroid hormones, even under the influence of MMI, as modern swine appear especially resistant to the action of antithyroid drugs (Fazioli et al. 2024).

The IGF ligands, *IGF1* and ‐*2*, experienced no changes in expression in response to MMI treatment, with only moderate ontogenic changes observed in the expression of *IGF1* among the CON groups. Canonically, the majority of IGFs are produced by the LVR; however, localized IGF expression and production have previously been noted in a wide array of fetal tissues (Camm et al. 2021; Latimer et al. 1993), consistent with the local IGF expression observed in the fetal KID in the present study. The present work reporting a lack of effect of hypothyroidism on *IGF1* expression is consistent with prior work in swine in which late gestation fetal hypothyroidism resulted in no alterations in circulating IGF1 levels (Spencer et al. 1989). This finding, however, contrasts with work in sheep showing that hypothyroidism leads to decreased adrenocortical and skeletal muscle *IGF1* expression (Camm et al. 2021; Forhead et al. 2002), suggesting either a tissue or species‐specific relationship between thyroid hormones and the IGF system. While IGF1 is known to be upregulated postnatally (Pan et al. 2012; Spencer et al. 1989; Lee et al. 1993), IGF2 is substantially upregulated in utero and concordantly thought to play a vital role in the control of fetal growth (Gluckman and Butler 1983; Näntö‐Salonen et al. 1991; Pan et al. 2012). Circulating IGF2 levels have previously been shown to be independent of thyroid status in the fetal lamb (Mesiano et al. 1989), supporting the lack of transcriptional change observed in the present study.

Tissue‐specific ontogenic alterations in expression of many of the IGFBPs were also observed throughout gestation in the present study, with expression of five of the assessed IGFBPs increased in the LVR at GD 86 relative to GD 55. While this is the first study to examine the transcriptional ontogeny of such a wide range of IGFBPs in the porcine fetus, our results are consistent with one prior report in the pig indicating increased hepatic *IGFBP2* expression throughout gestation (Lee et al. 1993), and also with another reporting increased hepatic *IGFBP3* expression during late gestation (Peng et al. 1996). In addition to ontogenic increases in hepatic IGFBP expression, the present study found that these transcriptional changes occurred in concurrence with increased sera IGFBP levels. This is consistent with that previously observed in porcine fetuses of a similar gestational age, in which levels of IGFBP2, ‐3, ‐4, and an unknown 29 kDa IGFBP were increasing in fetal porcine serum between GDs 50–75 (Hausman et al. 2000). IGFBPs have been speculated to serve many functions, including preventing the interaction of IGFs with their receptors, prolonging IGF half‐lives, sequestering IGFs in specific tissues, or even exerting IGF‐independent actions (Guler et al. 1989; LeRoith et al. 2021). While the exact biological significance of increased IGFBP levels throughout gestation would require more functional studies to further understand, one could hypothesize that without a concomitant increase in ligand expression throughout gestation, increased IGFBP levels may indicate decreased IGF bioavailability, and thus a reduced dependence on the IGF system for growth during late gestation. In contrast, all observed dysregulations in IGFBP expression in the hypothyroid fetuses were downregulations, which may indicate an increased proportion of free IGFs for exertion of biological activity in the respective tissues.

While instances of non‐nuclear receptor signaling have been demonstrated, canonically, the cellular response of thyroid hormones requires the presence of at least one of the major thyroid hormone receptors, which are encoded by the *THRA* and *THRB* genes (Cheng 2000). These receptors are known to be both spatially and developmentally regulated (Brent 2012), but their exact expression patterns are unknown in the porcine fetus. In the present study, we show that both the fetal LVR and KID express *THRA* and *THRB* between GDs 55–86, suggesting that, even early in gestation, these tissues have the capacity to respond directly to thyroid hormones. Consequently, the noted transcriptional dysregulations in the fetal IGF system may be a direct result of transcriptional control by thyroid hormones. Alternatively, studies in other species have suggested that some hypothyroid‐induced transcriptional modulations in the IGF system may instead be mediated indirectly through changes in other circulating hormones such as growth hormone (Näntö‐Salonen et al. 1993; Ramos et al. 2002). While not assessed in the current study, prior research in late gestation fetal swine reported no significant changes to circulating growth hormone levels in response to hypothyroidism (Spencer et al. 1989), suggesting that modulation of the IGF system in the fetal pig may not occur via this indirect mechanism. However, studies in a more controlled model, such as a relevant in vitro cell culture line, must be performed to further elucidate the exact mechanism by which thyroid hormones modulate the IGF system in porcine fetal tissues.

While our results revealed that both the fetal LVR and KID express *THRA* and *THRB*, gene expression of IGFBPs in the fetal LVR was much more perturbed by the impact of fetal hypothyroidism. Most notably, expression of the predominant fetal IGFBP, *IGFBP2*, was downregulated at GDs 55, 66, and 76 in the LVR. In the KID, only *IGFBP3* and the comparatively low‐affinity *IGFBP7* were downregulated, with dysregulation of *IGFBP3* occurring at GDs 55, 66, and 76 only. Along with the other observed changes in the LVR, this suggests a substantial disruption in the hepatic IGF system, and a relatively smaller disruption in the renal system, with both tissues displaying an increased resiliency to endocrine disruption as gestation progresses. A visual assessment of band intensity in our PCR results indicates a greater abundance of both major thyroid hormone receptors in the fetal KID in comparison to the LVR, which would not explain the tissue discrepancy in response to thyroid disruption observed in the present study. In addition to receptor abundance, however, the bioavailability of thyroid hormones may be regulated by various other factors including thyroid hormone binding protein abundance (Bartalena and Robbins 1993) and iodothyronine deiodinase (DIO) activity, the latter of which may be especially important in regulating tissue and cell‐specific levels of bioactive thyroid hormones (Luongo et al. 2019). One prior study in late gestation fetal swine reported that transcriptional abundance of *DIO1* was much greater than that of *DIO2* or *DIO3* in the fetal porcine LVR and KID, with *DIO1* abundance also greater in the KID than the LVR (Ison et al. 2022). Unlike DIO2 and DIO3, which specifically activate and deactivate thyroid hormones, respectively, DIO1 has the capacity to produce both bioactive and biologically inactive thyroid hormones (St Germain et al. 2009), and it is unclear which of these functions would prevail in the fetal LVR and KID. In late gestation, *DIO1* is known to be downregulated in the fetal LVR, but not the fetal KID, in response to hypothyroidism (Ison et al. 2023), which could help to explain the differential sensitivity of fetal tissues to thyroid hormones. However, a better understanding of tissue‐specific thyroid hormone metabolism throughout mid‐ to late gestation is needed to increase understanding of the tissue discrepancy observed in gene expression.

In addition to the discrepancy in response of the renal vs. hepatic IGFBPs, it is curious that expression of some of the IGFBPs was dysregulated by hypothyroidism, while others remained stable. The six high‐affinity IGFBPs, IGFBPs 1–6, have a highly conserved structure and are thought to have arisen via whole genome duplication events (Allard and Duan 2018). An in‐depth analysis of IGFBP structure has shown that IGFBPs ‐1, ‐2, and ‐4 are more closely related to each other than they are to IGFBPs ‐3, ‐5, and ‐6 (Daza et al. 2011). In the present study, assessment of these high‐affinity binding proteins revealed that hepatic expression of *IGFBP1*, ‐*2*, ‐*3*, and ‐*4*, and renal expression of *IGFBP3* were all significantly impacted by fetal hypothyroidism at least two of the studied time points. Interestingly, however, while *IGFBP3* was impacted by fetal hypothyroidism, there was minimal to no impact on expression of the highly related genes *IGFBP5* and *IGFBP6*. As thyroid hormones require short nucleotide sequences called thyroid hormone response elements (TREs) to exert their canonical transcriptional regulation (Cheng 2000), one possible explanation for this discrepancy may be the presence or absence of TREs upstream of the individual IGFBP genes. While an analysis of TREs in these genes in the pig is yet to be performed and is beyond the scope of the present study, such investigation would provide insights into the mechanism by which thyroid hormones may regulate transcriptional activity of the IGFBP genes. It is important to note, however, that accurate in silico identification of TREs is particularly challenging due to the wide variability in sequence that has previously been noted (Dudazy‐Gralla et al. 2013).

Consistent with prior work in the fetal pig, we found Western Ligand Blotting to allow for detection and relative quantification of IGFBP2, ‐3, and ‐4 (Hausman et al. 2000; Peng et al. 1996), but also detected two bands in the expected region for IGFBP1. While these bands may represent IGFBP1 and ‐5, they are unlikely to represent IGFBP6, as IGFBP6 has a much higher binding affinity for IGF2 in comparison to the IGF1 ligand utilized in the present study (Bach 2018). Many prior papers report detected bands within this unknown region to represent IGFBP1 alone (Latimer et al. 1993; Peng et al. 1996); however, one prior study reported that adipose tissue IGFBPs detected at this molecular weight range may be a combination of IGFBP1, ‐5, and glycosylated IGFBP4 (Hausman et al. 2000). As a result, elucidating the exact identity of these two bands would require further immunoblotting analyses, which may be difficult considering the limited availability of validated antibodies for the pig, and also the similarity in IGFBP structure that increases the probability of unintended antibody cross‐reactivity.

While many transcriptional alterations in IGFBP expression were observed in the fetal LVR and KID in response to hypothyroidism, there was a minimal impact of hypothyroidism on sera IGFBP levels as assessed by Western Ligand Blotting, with only a temporal decrease observed in the abundance of the unknown 28 kDa IGFBP at GD 76. A prior publication in swine found sera IGFBP levels in fetuses rendered hypothyroid by hypophysectomy to be temporally responsive to fetal thyroid status (Latimer et al. 1993), although it is important to note that hypophysectomy disrupts levels of many hormones regulated by hypothalamic–pituitary action, and certainly not just thyroid hormones. In contrast to prior results, the minimal impact of hypothyroidism on sera IGFBP levels in the more controlled model of thyroid disruption utilized in the present study suggests that the systemic IGF system is not substantially disrupted by hypothyroidism in the fetal pig. Additionally, when interpreted in conjunction with prior studies, our results may suggest that systemic IGFBP production is partly under the regulation of another endocrine pathway, which could have contributed to results historically observed in hypophysectomised fetuses.

## 5 Conclusion

Collectively, the results of the present study demonstrate that both thyroid hormone levels and components of the IGF system are temporally regulated throughout gestation in the fetal pig. Further, they demonstrate a complex temporal and tissue‐specific interaction between the porcine fetal thyroid and IGF system, with hypothyroidism having a more sizable impact on the IGF system within the fetal LVR in comparison to the fetal KID. Expression of the major IGF ligands, *IGF1* and *IGF2*, was not altered by MMI‐induced hypothyroidism, but six out of seven of the assessed IGFBPs were temporally dysregulated in the hypothyroid fetal LVR, with two of the IGFBPs temporally dysregulated in the fetal KID. While these results support a relationship between thyroid hormones and the fetal IGF system, the magnitude of the observed dysregulations in both tissues was modest, suggesting that the importance of thyroid hormones in regulating this system may be relatively low. Additionally, hypothyroidism had minimal impact on levels of circulating IGFBPs in fetal serum, suggesting that porcine fetal hypothyroidism causes secondary endocrine disruption by temporally dysregulating tissue expression of the IGFBPs, but this is not marked by concomitant, systemic dysregulations in the protein level of the IGFBPs. Future research should examine the prolonged impact of congenital hypothyroidism on the IGF system to determine if fetal modulation of this system may impact postnatal physiology and phenotype through in utero programming.

## Author Contributions

J. Alex Pasternak and Alyssa A. Smith conceived of the study and initial hypothesis. Alyssa A. Smith conducted the laboratory procedures, performed the statistical analyses, and drafted the manuscript, which was reviewed and approved by J. Alex Pasternak.

## Ethics Statement

All animal procedures were carried out in compliance with Purdue University's animal care policies and approved by the Institutional Animal Care and Use Committee (IACUC Protocol #0123002344).

## Conflicts of Interest

The authors declare no conflicts of interest.

## Supporting information


Data S1.

## Data Availability

The datasets generated during the current study are available from the corresponding author upon reasonable request.
