# Supplementary material for: Porcine Fetal Hypothyroidism Induces Temporal and Tissue‐Specific Alterations in the Insulin‐Like Growth Factor System
Source: Compr Physiol. 2025 Jul 22;15(4):e70028. doi: 10.1002/cph4.70028 (PMC12281263; doi:10.1002/cph4.70028)
Supplement: Supplementary file 1 — Data S1. [file CPH4-15-e70028-s001.docx]

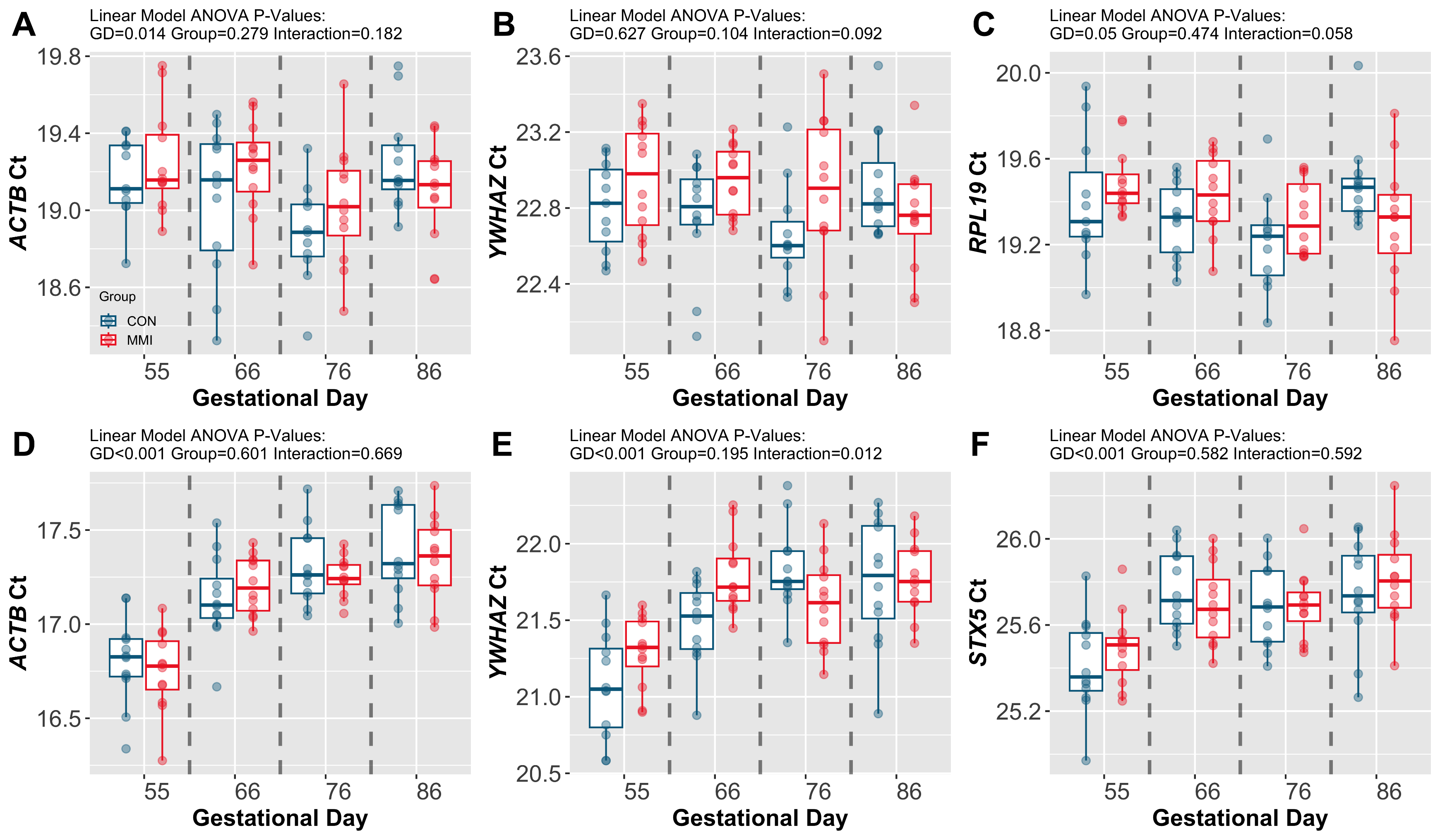


Supplemental Figure S1: *Changes in RT-qPCR reference gene expression.* Boxplots showing raw Ct values of RT-qPCR reference genes in fetal (A, B, C) liver and (D, E, F) kidney tissue derived from fetuses (*n* = 11-12/group) at various gestational timepoints following 21 days of maternal CON or MMI treatment. Stability was assessed using a linear model including gestational day (GD), treatment group, and the interaction, and the associated *P*-values listed above each figure panel.


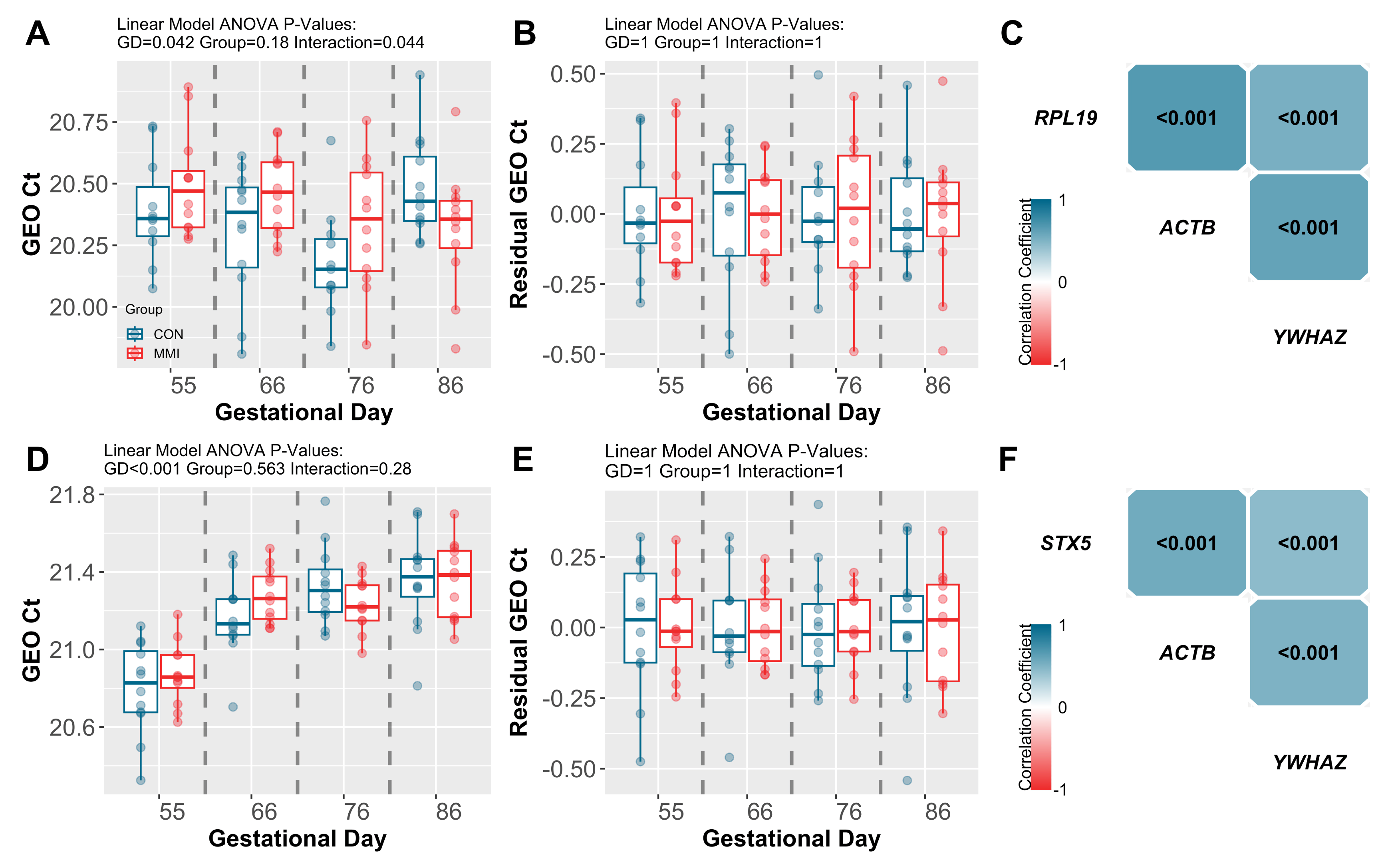


Supplemental Figure S2: *Normalization of RT-qPCR data.* Boxplots comparing stability of the geometric means (GEOs) of reference gene raw Ct values in fetal (A) liver and (D) kidney tissue derived from fetuses (*n* = 11-12/group) at various gestational timepoints following 21 days of maternal CON or MMI treatment, versus the stability of the GEOs of the residual Ct values in the fetal (B) liver and (E) kidney. Stability was assessed using a linear model including gestational day (GD), treatment group, and the interaction, with the associated *P*-values listed above each figure panel. The residual Ct values for each reference gene were determined to be highly correlated in both the fetal (C) liver and (F) kidney, indicating that the corresponding GEOs are representative of, and able to correct for, technical error.


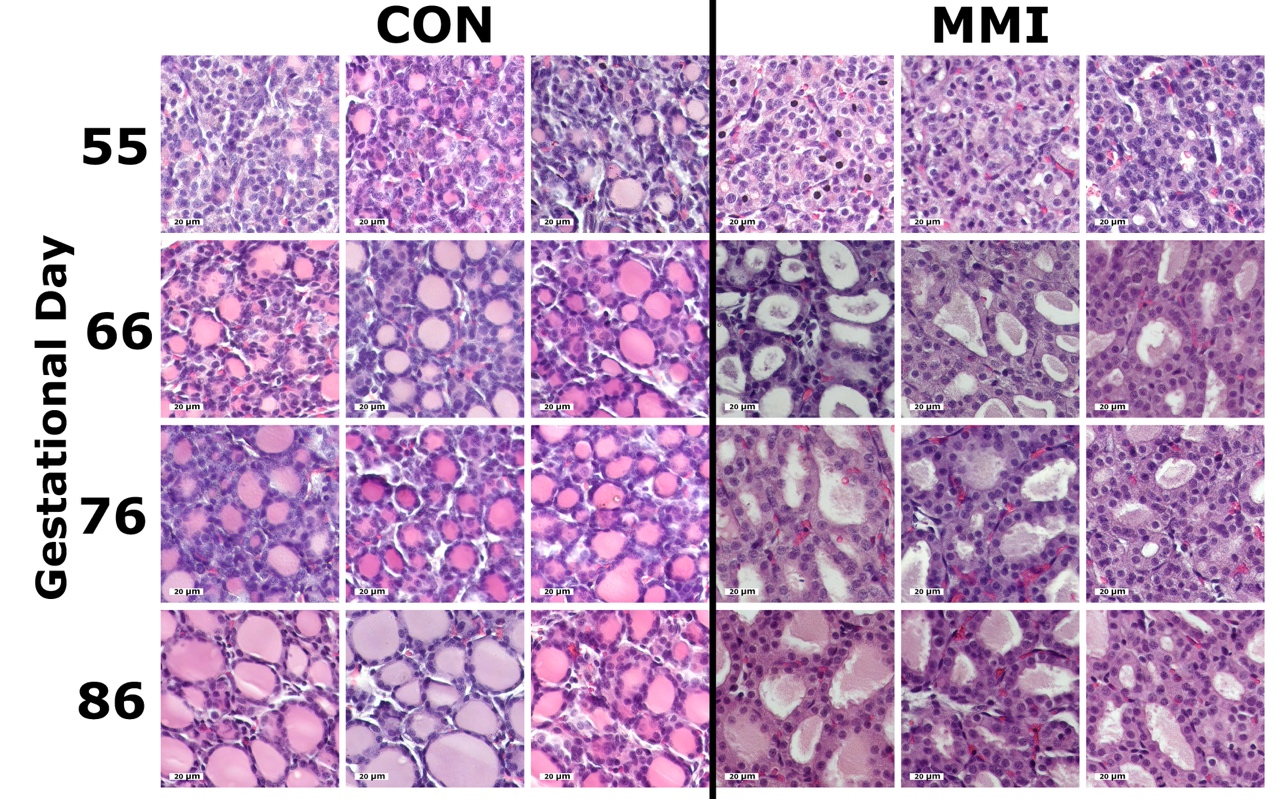


Supplemental Figure S3: *Fetal thyroid histology.* Representative images of fetal thyroid histology following 21 days of maternal CON or MMI treatment, with the 24 images representing one fetus from each of the 24 litters utilized in this study.
